# The Effect of Oxidative Stress Towards The Expression of Thiamine Biosynthesis Genes (THIC and THI1/THI4) in Oil Palm (*Elaeis guineensis*)

**DOI:** 10.21315/tlsr2018.29.1.5

**Published:** 2018-03-02

**Authors:** Zainor Hafisah Che Idris, Aisamuddin Ardi Zainal Abidin, Atiqah Subki, Zetty Norhana Balia Yusof

**Affiliations:** Department of Biochemistry, Faculty of Biotechnology and Biomolecular Sciences, Universiti Putra Malaysia, 43400 UPM Serdang, Selangor, Malaysia

**Keywords:** Thiamine, Oxidative Stress, Oil Palm, Gene Expression

## Abstract

Thiamine is known to be an important compound in human diet and it is a cofactor required for vital metabolic processes such as acetyl-CoA biosynthesis, amino acid biosynthesis, Krebs and Calvin cycle. Besides that, thiamine has been shown to be involved in plant protection against stress. In this study, the level of expression of THIC and THI1/THI4, the genes for the first two enzymes in the thiamine biosynthesis pathway were observed when oil palm (*Elaeis guineensis*) was subjected to oxidative stress. Primers were designed based on the consensus sequence of thiamine biosynthesis genes obtained from *Arabidopsis thaliana*, *Zea mays*, *Oryza sativa*, and *Alnus glutinosa*. Oxidative stress were induced with various concentrations of paraquat and samplings were done at various time points post-stress induction. The expression of THIC and THI1/THI4 genes were observed via RT-PCR and qPCR analysis. The expression of THIC was increased 2-fold, while THI1/THI4 gene transcript was increased 4-fold upon induction of oxidative stress. These findings showed that oil palm responded to oxidative stress by over-expressing the genes involved in thiamine biosynthesis. These findings support the suggestion that thiamine may play an important role in plant protection against stress.

## Abstract (ru)

Tiamina dikenali sebagai sebatian penting dalam diet manusia dan ia adalah kofaktor yang diperlukan untuk proses metabolisme yang penting seperti biosintesis asetil- CoA, biosintesis asid amino, dan juga kitaran Krebs dan Calvin. Selain itu, tiamina juga telah terbukti terlibat dalam perlindungan tumbuhan terhadap tekanan. Dalam kajian ini, tahap ekspresi transkrip gen THIC dan THI1/THI4, dua enzim pertama dalam tapakjalan biosintesis tiamina telah dianalisa apabila anak pokok kelapa sawit didedahkan kepada tekanan oksidatif. Primer direka berdasarkan perbandingan gen biosintesis tiamina daripada *Arabidopsis thaliana, Zea mays, Oryza sativa* dan *Alnus glutinosa*. Tekanan oksidatif telah diberikan melalui parakuat dengan pelbagai kepekatan dan sampel telah diambil di beberapa peringkat masa selepas induksi tekanan. Ekspresi transkrip gen THIC dan THI1/THI4 telah dianalisa melalui RT-PCR dan qPCR. Ekspresi transkrip gen THIC telah meningkat sebanyak dua gandaan, manakala ekspresi transkrip gen THI1/THI4 telah meningkat sebanyak empat gandaan selepas induksi tekanan oksidatif diberikan. Penemuan ini menunjukkan bahawa kelapa sawit bertindak balas kepada tekanan oksidatif dengan lebih-meningkatkan ekspresi gen-gen yang terlibat dalam biosintesis thiamine. Penemuan ini menyokong cadangan bahawa tiamina boleh memainkan peranan penting dalam perlindungan tumbuhan terhadap tekanan.

## INTRODUCTION

Thiamine (vitamin B1) is essential to human because it can not be naturally synthesised by human metabolism. However, the presence of thiamine, in our diet is very important because it acts as cofactor for metabolic processes such as the biosynthesis of acetyl-CoA and amino acids as well as the Krebs and Calvin cycles (Pourcel *et al.* 2013; Tunc-Ozdemir *et al*. 2009). The active form is known to be thiamine pyrophosphate (TPP) (Pourcel *et al*. 2013; Tunc-Ozdemir *et al*. 2009). Thiamine has also been found to play an important role as a stress-response molecule. Upregulation of thiamine biosynthesis genes and overproduction of thiamine have been demonstrated when stress is present, be it abiotic or biotic (Balia Yusof *et al*. 2015; Tunc-Ozdemir *et al*. 2009).

Oxidative stress is classified as abiotic and is triggered by environmental factors which can cause a series of changes within plants. It have been observed that oxidative stress negatively affect plant growth, development, and productivity by generating morphological, physiological, biochemical and molecular changes (Kumar & Venkateswarlu 2011).

Oil palm is the main crop of Malaysia and it is the main producer of palm oil and palm kernel oil. Palm oil is versatile since can be used for food and non-food purposes and it acts the cheapest traded edible oil (Page & Lord 2006; Oil 2009). In 2014, Malaysia produced about 85% of the world’s palm oil (Sime Darby 2014).

### Thiamine Biosynthesis

The thiamine biosynthesis pathway in all three kingdoms are highly conserved with two separate branches synthesizing the thiazole and pyrimidine moieties. These moieties are then coupled to form thiamine monophosphate (TMP) and the latter is then phosphorylated to form thiamine diphosphate (TDP), also known as thiamine pyrophosphate (TPP), which acts as an active cofactor (Bettendorff 2007; Croft *et al*. 2006). However, the precursors for the biosynthesis differ between plants and microorganisms. In bacteria, the precursors are derived from purine and isoprenoid biosynthesis, while in yeast, they are derived from vitamin B_3_ and B_6_ compounds (Pourcel *et al*. 2013). Unlike bacteria and yeast, plant biosynthesis pathway involves the combination of both.

**Figure 1:**
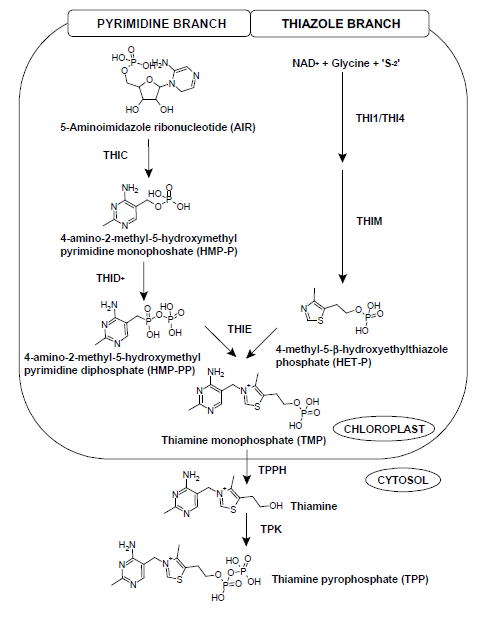
The thiamine biosynthesis pathway which consists of pyrimidine branch and thiazole branch (Croft *et al*., 2006). Each branch moiety will be condensed together by the action of TMP synthase to produce thiamine monophosphate (TMP). This reaction occurs in the chloroplast. By the reaction of TPPH, thiamine is produced in cytosol. Thiamine pyrophosphokinase (TPK) will synthesise thiamine into the active cofactor, TPP.

Thiamine biosynthesis in plants begins with the synthesis of pyrimidine and thiazole moieties from 5-aminoimidazole ribonucleotide (AIR) and NAD^+^, glycine, and sulfur respectively (Figure 1). To synthesise the pyrimidine moiety, AIR is converted to 4-amino-2-methyl-5-hydroxymethylpyrimidine monophosphate (HMP-P) by HMP-P synthase (THIC). This pyrimidine moiety synthesis is similar to the one occurring in bacteria rather than in yeast (Rapala-Kozik *et al*. 2008; Pourcel *et al*. 2013). HMP-P is phosphorylated to 4-amino-2-methyl-5-hydroxymethylpyrimidine diphosphate (HMP-PP) by HMP-P kinase (THID) (Rapala-Kozik *et al*. 2008). THID is a bifunctional protein which has been characterised in *Zea mays* as THI3 and in *Arabidopsis thaliana* as THI1 (Ajjawi *et al*. 2007; Rapala-Kozik *et al*. 2007; Pourcel *et al*. 2013). THID, a single, two-domain protein (Rapala-Kozik *et al*. 2008) also catalyzes the phosphorylation of HMP-PP to thiamine monophosphate (TMP). The enzyme is also known as TMP synthase encoded by the gene THIE (Rapala-Kozik *et al*. 2008; Pourcel *et al.* 2013). Interestingly in plants, the thiazole ring, 4-methyl-5-β-hydroxyethylthiazolephosphate (HET-P), formation is said to be similar in yeast (Chatterjee *et al*. 2007; Rapala-Kozik *et al*. 2008; Chatterjee *et al*. 2011; Pourcel *et al.* 2013). Precursors namely NAD^+^, glycine, and sulfur (from a backbone of cysteine in THI4p) were catalyzed by HET-P synthase (THI1/THI4) and subsequently THIM to produce HET-P. THI4p is a HET-P synthase known in yeast (Pourcel *et al*. 2013), while THI1 is a HET-P synthase known in *Arabidopsis thaliana* (Rapala-Kozik *et al*. 2008). The THI4 enzyme and its homolog, THI1, has another function other than its involvement in thiamine biosynthesis. It functions as a component involved in mitochondrial DNA damage tolerance (Machado *et al*. 1997), against heat stress (Ferreira *et al*. 2006; Tunc-Ozdemir *et al*. 2009), and also disease resistance (Wang *et al*. 2006; Tunc-Ozdemir *et al*. 2009). HMP-PP and HET-P are then condensed together by TMP synthase (THIE) to produce TMP. All of these occur in the chloroplast (Pourcel *et al*. 2013).

TMP is dephosphorylated by the enzyme phosphatase (TPPH) to free thiamine and this reaction takes place in the cytosol (Pourcel *et al*. 2013). The pyro-phosphorylation of thiamine to thiamine pyrophosphate (TPP) occurs by the reaction of thiamine pyrophosphokinase (TPK). Croft *et al.* (2007) stated that prokaryotes could convert TMP to TPP via the *thiL* gene product. However in eukaryotes, dephosphorylation of TMP to thiamine occurs before the phosphorylation of thiamine to form TPP which is catalysed by TPK. Thiamine biosynthesis pathways in *Arabidopsis thaliana* and *Zea mays* have similarities with *Chlamydomonas reinhardtii.* In *C. reinhardtii*, the formation of the thiazole moiety, HET-P from NAD^+^, glycine, and a sulfur donor, is catalysed by THI4 as the first committed step. The pyrimidine moiety, HMP-PP is produced by AIR which is an intermediate in histidine and purine biosynthesis catalysed by THIC and THID enzyme (Croft *et al*. 2007). Further reaction is the phosphorylation and pyrophosphorylation of TMP to thiamine and finally, TPP by phosphatase and TPK, respectively.

## MATERIALS AND METHODS

### Plant Materials and Stress Treatment

A total of 12 six-month old commercial Dura × Pisifera (DXP) oil palm seedlings were obtained from Sime Darby, Banting. The seedlings were arranged in a shaded area using randomised complete block design (RCBD) and general nursery practices were carried out. Three seedlings were used for each parameter namely control (no treatment), 0.006M, and 0.009M paraquat concentrations (Hextar Paraquat 13).

### Sampling

Sampling was carried out at 3-, 7- and 30-days post-treatment. Spear leaves were taken as tissue samples from 4 individual seedlings at each sampling. All spear leaves were taken, cleaned and cut before being frozen in liquid nitrogen and kept in −80°C for further use.

### Data Mining and Primer Designing

Primers for the amplification of THIC and THI1/THI4 gene fragments were designed from the consensus sequences obtained from multiple sequence alignment of sequences of genes obtained from various plant species. For quantitative polymerase chain reaction (qPCR), primers were designed based on sequence obtained from the amplified fragments. Table 1 shows all the primers designed and used in this study.

### Total RNA Isolation and Quantitation

Total RNA was isolated using modified RNA extraction protocol by Li and Trick (2005). The tissue used was oil palm spear leaves. Assessments of RNA concentration and purity were done spectrophotometrically (NanoPhotometer Implen, Germany). The RNA samples were kept at −80°C until further use.

### Amplification of THIC and THI1/THI4 Genes

Reverse transcriptase polymerase chain reaction (RT-PCR) was performed using Tetro cDNA Synthesis Kit (Bioline, USA). Complementary DNA (cDNA) was synthesised by mixing 5 μg/μl of total RNA, 4 μl of 5× Reverse Transcriptase Buffer, 1 μl of 10 mM dNTP mix, 1 μl of Oligo (dT)_18_ Primer, 1 μl of Ribosafe RNase Inhibitor, 1 μl of Tetro Reverse Transcriptase and DEPC-treated water to make up to 20 μl. The reaction mixture was homegenised by pipetting gently and incubated at 45°C for 30 minutes. This was followed by an incubation at 85°C for 5 minutes to stop the reverse transcriptase reaction and the holding temperature was finally held at 4°C. The cDNA was kept −20°C until further use.

PCR was performed by using MyTaq^TM^ Red Mix (Bioline, USA). A 25 μl reaction mixture was prepared by mixing 1 μl of cDNA template, 0.5 μl of 10 μM forward primer, 0.5 μl of 10 μM reverse primer, 12.5 μl of MyTaq Red Mix and deionised water to make up to 25 μl**.** The mixture was mixed gently and placed inside the thermocycler (Biometra, Germany). The PCR cycling conditions involved an initial denaturation step set at 95°C for 2 minutes, followed by 28 repetitive cycles of denaturation step at 95°C for 45 seconds, annealing step at 55°C for 45 seconds and extension step at 72°C for 1 minute. The final cycle of extension was set at 72°C for 5 minutes and then held at 4°C. The PCR product was then kept at −20°C until further use.

### Analysis of PCR Products

PCR products were analysed using gel electrophoresis and the intensity of the bands of the amplified gene fragments were calculated using ImageJ software (http://imagej.nih.gov/ij/).

### PCR Product Purification

The PCR products were purified using FavorPrep™ Gel/PCR Purification Kit (Favorgen). 100 μl of PCR products were transferred in to a microcentrifuge tube and mixed with 500 μl of FADF buffer by vortexing for 12 minutes. The mixture was then transferred into FADF column in a collection tube and centrifuged for 30 seconds at 16,000 × g (Heraeus, Germany). The flow-through was discarded and 750 μl of wash buffer was added into the FADF column and centrifuged briefly for 30 seconds and followed by another further 3 minutes at 16,000 × g. The column was then placed into a new microcentrifuge tube and the purified PCR product was eluted out by adding 30 μl of elution buffer to the centre of the column followed by centrifugation for 2 minutes at 16,000 × g after a 2 minute stand at room temperature. Each purified PCR product was analysed with a nano-spectrophotometer (Implen, Germany) for DNA purity and concentration prior sending for sequencing.

### DNA Sequencing

Purified PCR products were sent for sequencing to 1st BASE DNA sequencing service centre (1st BASE, Singapore). The FASTA sequences obtained were then analysed using Basic Local Alignment Search Tool (BLAST) (https://blast.ncbi.nlm.nih.gov) for further analysis.

### qPCR Analysis

A qPCR analysis was performed using SensiFAST™ SYBR No-ROX Kit (Bioline). 0.3 μg of cDNA template was mixed with 0.4 μl of 10 μM forward qPCR primer, 0.4 μl of 10 μM reverse qPCRprimer, 10 μl of 2 × SensiFast SYBR No-ROX Mix (containing 10 μM of dNTP mixture, 3 mM of MgCl_2,_ SYBR® Green I dye, *Taq* Polymerase buffer, *Taq* DNA Polymerase, stabilisers and enhancers) and 8.2 μl of PCR grade distilled water. Each mixture was pipetted into a 0.2 ml PCR tube, vortexed and centrifuged for a short spin prior to placing it into the Rotor-Gene Q thermocycler (QIAGEN, Germany). Two step qPCR was performed with the cycling conditions consisting of an initial denaturation step at 95°C for 2 minutes, 40 cycles of denaturation (95°C for 5 seconds) and 65°C of annealing reaction. Each reaction was done in triplicate and the expressions of the genes of interest were analysed against the reference housekeeping gene, GADPH.

## RESULTS AND DISCUSSION

### Primer Designing and Amplification

Primers for RT-PCR were designed based on a multiple sequence alignment analysis of genes from different plant species while qPCR primers were designed based on the amplified gene fragments sequence. Table 1 shows all the primers designed and used in this study. For THIC gene fragment, two pairs of primers were designed and fragments with the size of 410 basepairs and 156 basepairs were successfully amplified using primer pairs F2 and F3′ respectively. For THI1/THI4 gene fragment, a fragment of 180 basepair was successfully amplified using primer pair F8.

The amplified gene fragments were sequenced and primers for qPCR were designed based on the results obtained. For the amplification of THIC gene fragment, a pair of primers (designated as THIC F3 primers) managed to amplify a 188 basepair fragment while for THI1/THI4 gene fragment, the pair designated as THI4 F3 primers managed to amplify a 104 basepair fragment. A pair of primers designated as GADPH F1 primers were designed to amplify a housekeeping gene, GADPH and they managed to amplify a fragment with the size of 110 basepair.

### Sequencing Analysis

Prior to the designing of qPCR primers, purified PCR products of amplified gene fragments were sequenced using 1st BASE DNA sequencing service and the FASTA sequences were then analysed using BLAST. For the amplified THIC gene fragment, the sequencing result shows 98% sequence similarity to *Elaeis guineensis* phosphomethylpyrimidine synthase, chloroplastic (LOC105046270), transcript variant X10, mRNA (Accession number: XM_010924818.1). The E value was 1e-72. THI1/THI4 gene fragment amplified showed 99% sequence similarity to PREDICTED: *Elaeis guineensis* probable thiamine biosynthetic bifunctional enzyme, mRNA (Accession number: XM_010932306.1). On the other hand, amplified GADPH gene fragments shows 92% sequence similarity to PREDICTED: *Elaeis guineensis* glyceraldehyde-3-phosphate, mRNA (Accession number: XM_010944010.1).

### Expression of THIC and THI1/THI4 Genes under Oxidative Stress

**Figure 2:**
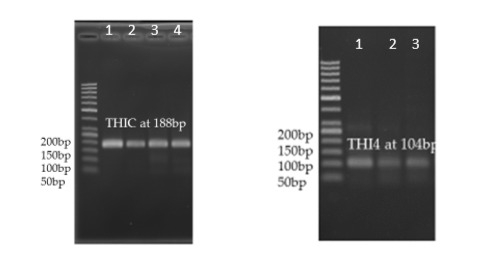
The amplification of (A) THIC gene and (B) THI4 gene from oil palm spear leaves. M represents the VC 100bp DNA marker. Lanes 1, 2, 3 and 4 for (A) represents cDNA samples from control, 0.003 M of paraquat, 0.006 M of paraquat and 0.009 M of paraquat at Day 3. Lanes 1, 2 and 3 for (B) represents cDNA samples from control, 0.006 M of paraquat and 0.009 M of paraquat at Day 3.

**Figure 3:**
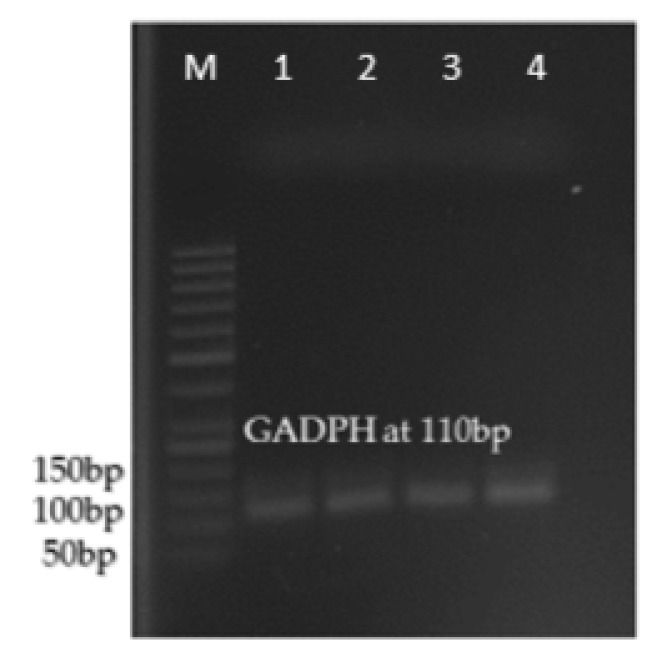
The amplification of GADPH from oil palm spear leaves. M represents the VC 100bp DNA marker. Lanes 1, 2, 3 and 4 represents cDNA samples from control, 0.003 M of paraquat, 0.006 M of paraquat and 0.009 M of paraquat at Day 3.

The expression of THIC and THI1/THI4 genes were quantified using the relative quantification approach where the expression of gene of interest was analysed against expression of the housekeeping gene, GADPH as described by Pfaffl, (2004). Both THIC and THI1/THI4 gene fragments were successfully amplified from both treated and non-treated tissue samples (Fig. 2). Amplification of the cDNA expressed housekeeping gene, GADPH was also successful and shown visually in Figure 3.

For the expression analysis, two analyses were carried out. Based on the results obtained from the RT-PCR analysis, the difference in the level of expressions could be seen in which there was an increase in the paraquat-treated tissue samples for both gene transcripts as shown in Table 2. The non-treated seedlings act as a control which is presented as value one. For paraquat-treated seedlings, the intensity of the amplified gene transcripts showed an increase which suggested the upregulation of the expression of both thiamine biosynthesis genes, THIC and THI1/THI4, upon stress application.

**Figure 4:**
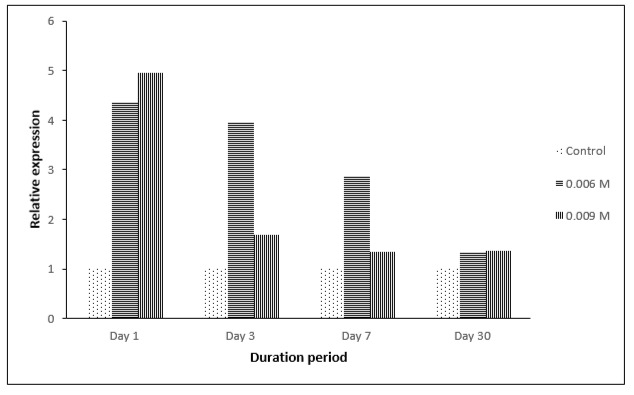
qPCR analysis for THI1/THI4 gene expression in oil palm spear leaves under oxidative stress at different duration period.

**Figure 5:**
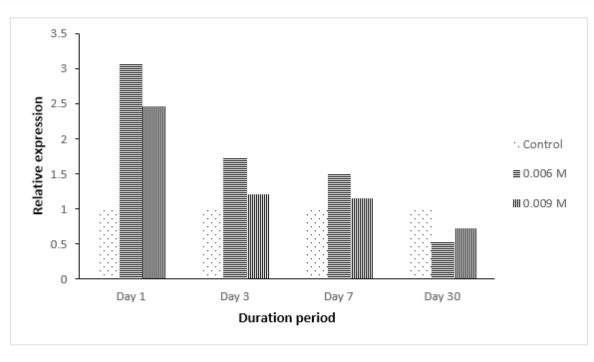
qPCR analysis for THIC gene expression in oil palm spear leaves under oxidative stress at different duration period.

However, based on qPCR analysis (Fig. 4 and 5), at all concentrations of paraquat (PQ) given (0.006M and 0.009M), upregulation of THIC and THI1/THI4 genes transcripts were observed especially on day one. For THIC gene, the highest upregulation was observed for PQ 0.006 M, day one post-treatment (Fig. 4). In general, for any given concentration, the expression levels were shown to be increasing up to a point and then started to decrease over time. For 30 days post-treatment, most of the samples showed no upregulation of the THIC gene transcript. This might be due to the fact that the seedlings had adapted to the condition applied or the plant was exercising another mechanism to cause such change.

For THI1/THI4 gene, it was observed that the highest expression was detected for PQ 0.009 M, day one post-treatment (Fig. 5). The same pattern was observed for PQ 0.006 M. For 30 days post-treatment, a slight upregulation in THI1/THI4 gene transcript was observed for PQ 0.009 and PQ 0.006 M. This could be due THI1/THI4 enzyme having another function other than being involved in thiamine biosynthesis, such as playing a role in DNA damage repair.

The results obtained showed that there was an upregulation in THIC and THI/THI4 gene transcription in the earlier stage specifically at day one with up to more than 2-fold of increase after the exposure to stress. This indicated that thiamine was needed to be synthesised in order to combat the damaging effect of osmotic stress. This is not surprising as many studies have suggested that thiamine may be involved as a response molecule towards abiotic stresses in plants (Ribeiro *et al.* 2005; Rapala-Kozik *et al.* 2012). However, the decrease of the transcription level of THIC and THI1/THI4 gene transcripts along the period of stress treatment could be due to the fact that the palms are starting to adapt to the stress conditions. There is yet any proof of thiamine being directly involved in combating stresses, but thiamine is known to have major roles in carbohydrate catabolism, NADPH and ATP synthesis and in the formation of nucleic acids (Rapala-Kozik *et al.* 2012) which are indirectly involved in the production of compounds involved in defence mechanism.

## CONCLUSION

In this study, THIC and THI1/THI4 primers have been successfully designed and the gene fragments coding for the enzyme (HMP-P synthase (THIC) and HET-P synthase (THI1/THI4)) were amplified and sequenced from oil palm (*Elaeis guineensis*). Also, the expression levels of THIC and THI1/THI4 gene transcripts showed an increase when subjected to oxidative stress induced by paraquat. The expression of THIC and THI1/THI4 gene transcripts were at the highest at day one post-treatment in all of the concentrations given. These results suggest that the upregulation of the first two enzymes in thiamine biosynthesis pathway when the plants were subjected to oxidative stress may be due to thiamine’s function in plant protection. These findings however need to be further verified but it is shedding some light on thiamine’s role in plant protection against stress in oil palm. Analysis on other enzymes involved in thiamine biosynthesis pathway will be further elucidated and the total thiamine content will be measured. It is hoped that this work will pave the way in understanding thiamine biosynthesis pathway in oil palm and its role in plant protection against stress.

## Figures and Tables

**Table 1:** Primer sequences used in this study together with its reference in designing the primers.

Primer	Reference sequence	Primer sequence
**THIC F2**	*Arabidopsis thaliana* phosphomethylpyrimidine synthase mRNA, complete cds (NM_001202705.1); *Arabidopsis thaliana* phosphomethylpyrimidine synthase mRNA, complete cds (NM_128517.3); *Arabidopsis thaliana* phosphomethylpyrimidine synthase mRNA, complete cds (NM_179804.2); *Zea mays* clone 378320 thiamine biosynthesis protein thiC mRNA, complete cds (EU972242.1); *Oryza sativa* Japonica Group Os03g0679700 (Os03g0679700) mRNA, complete cds (NM_001057432.1)	Forward 5′ - CTTACAGCAAAGAGAATGAC - 3′ Reverse 5′ - GTGATGTGATCATAACCAGG - 3′
**THI1/THI4 F8**	*Arabidopsis thaliana* thiazole biosynthetic enzyme mRNA, complete cds (NM_124858.3); *Arabidopsis thaliana* Thi1 protein mRNA, complete cds (U17589.1); *Zea mays* thiamine biosynthesis1 (thi1), mRNA (NM_001112226.1); *Oryza sativa* Japonica Group mRNA for thiamine biosynthetic enzyme, complete cds, clone: 12YPR001 (AB110170.1); *A. glutinosa* mRNA for thiazole biosynthetic enzyme (X97434.1)	Forward 5′- GACGCTATTGTGCGGTTGAC -3′ Reverse 5′ - TCCGTCAATAGCATTCGGCA - 3′
**GADPH F1**	*P.hortense* GADPH mRNA for glycolytic glyceraldehyde-3-phosphate dehydrogenase (205481:36–1046); *Cryptocercus punctulatus* glyceraldehyde-3-phosphate dehydrogenase mRNA, complete cds (JQ686947.1); *M. liliiflora* GADPH mRNA for glycolytic glyceraldehyde-3-phosphate dehydrogenase (19565:43–1068)	Forward 5′- GTCCCACCTGCTCAAGTACG- 3′ Reverse 5′ - CGGACACGACCTTGATGACC- 3′
**THIC F3**	Sequencing results	Forward 5′ - AATGAAGGTCCAGGGCAT - 3′ Reverse 5′ - GCTGAGGTGATGTGATCA - 3′
**THI1/THI4 F3**	Sequencing results	Forward 5′ - CCTTGCTGGCTATCGGGAATG- 3′ Reverse 5′ - ATGGCATCTGACGAATCTGAG- 3′
**GADPH F1**	Sequencing results	Forward 5′ - GTCCCACCTGCTCAAGTACG- 3′ Reverse 5′ - CGGACACGACCTTGATGACC- 3′

**Table 2:**
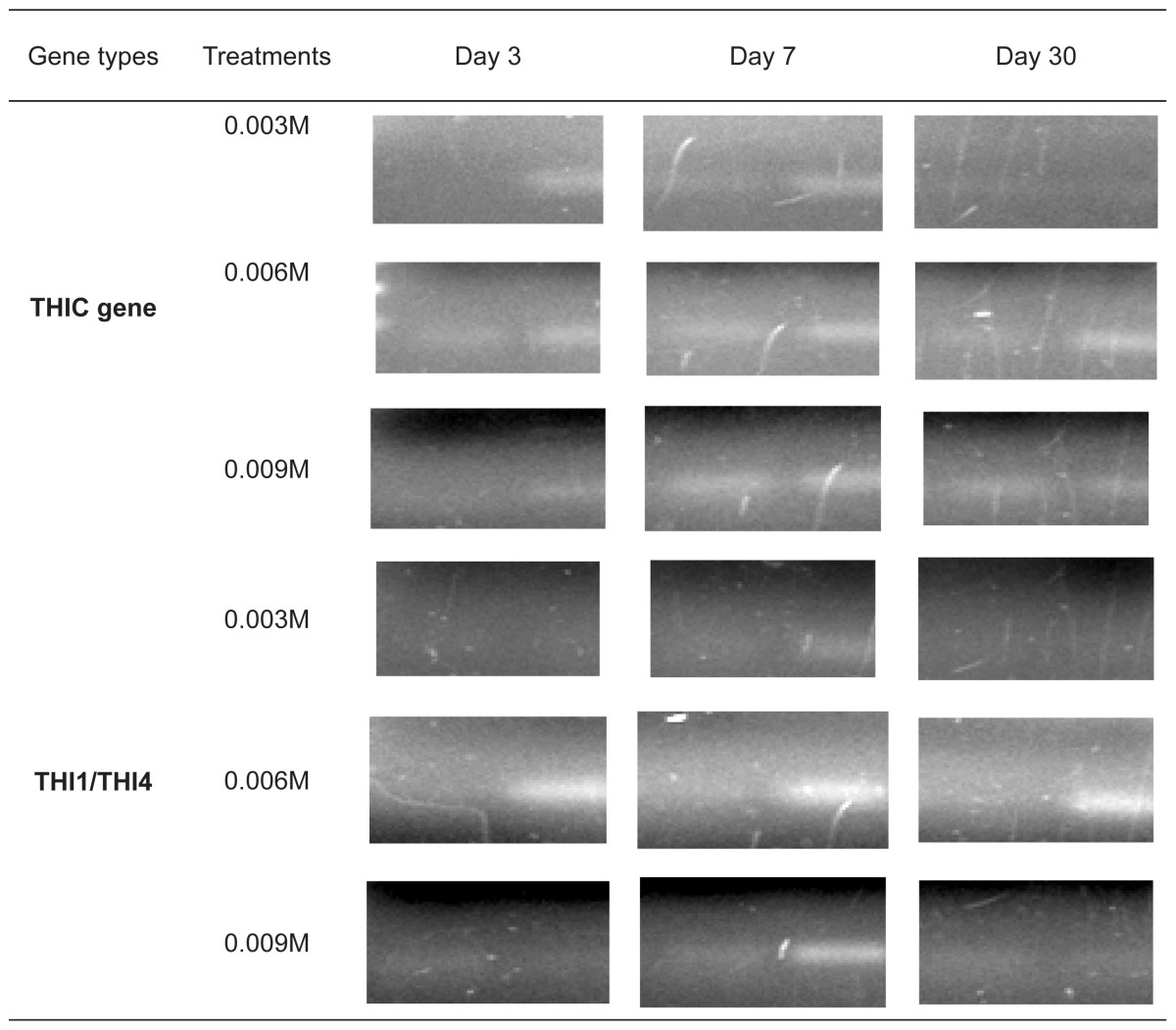
The comparison of intensity between (a) control spear leaf with (b) paraquattreated spear leaf of oil palm. The expression level or the intensity of the band, THIC and THI1/THI4 gene transcripts were measured by imageJ software.
